# An Alternative Approach to Analyze Ipsative Data. Revisiting Experiential Learning Theory

**DOI:** 10.3389/fpsyg.2015.01742

**Published:** 2015-11-13

**Authors:** Joan M. Batista-Foguet, Berta Ferrer-Rosell, Ricard Serlavós, Germà Coenders, Richard E. Boyatzis

**Affiliations:** ^1^Department of People Management and Organization, ESADE, Universitat Ramon LlullBarcelona, Spain; ^2^Department of Economics, University of GironaGirona, Spain; ^3^Organizational Behavior Department, Case Western Reserve UniversityCleveland, OH, USA

**Keywords:** ipsative data, Kolb's Learning Style Inventory (KLSI), compositional data analysis (CODA), emotional and social competencies, philosophical orientation

## Abstract

The ritualistic use of statistical models regardless of the type of data actually available is a common practice across disciplines which we dare to call type zero error. Statistical models involve a series of assumptions whose existence is often neglected altogether, this is specially the case with ipsative data. This paper illustrates the consequences of this ritualistic practice within Kolb's Experiential Learning Theory (ELT) operationalized through its Learning Style Inventory (KLSI). We show how using a well-known methodology in other disciplines—compositional data analysis (CODA) and log ratio transformations—KLSI data can be properly analyzed. In addition, the method has theoretical implications: a third dimension of the KLSI is unveiled providing room for future research. This third dimension describes an individual's relative preference for learning by prehension rather than by transformation. Using a sample of international MBA students, we relate this dimension with another self-assessment instrument, the Philosophical Orientation Questionnaire (POQ), and with an observer-assessed instrument, the Emotional and Social Competency Inventory (ESCI-U). Both show plausible statistical relationships. An intellectual operating philosophy (IOP) is linked to a preference for prehension, whereas a pragmatic operating philosophy (POP) is linked to transformation. Self-management and social awareness competencies are linked to a learning preference for transforming knowledge, whereas relationship management and cognitive competencies are more related to approaching learning by prehension.

## Introduction

When researchers use statistics for decision making, at least three different error types are usually identified. Type 1 and 2 errors are well-defined. Type 3 errors have been associated with rejecting the null hypothesis for the wrong reason (Mosteller, 1948), to incorrectly inferring the direction of the effect (Kaiser, 1960; Leventhal and Huynh, 1996) or even to giving the right answer to the wrong question (Kimball, 1957; Mitroff and Betz, 1972).

As far as we know, there is no error name yet for a more basic and ubiquitous mistake often made by practitioners who ritualistically use inappropriate statistical models or analysis techniques regardless of the type of data actually available. Examples of this includes, neglecting the violation of their statistical assumptions (e.g., non-normal bounded distributions) and of their substantive assumptions. The latter (violation of the substantive assumptions) ranges from misleading the nature of the factor measurement model specification—reflective vs. formative; or from omitting factor analysis model dimensions; to the omission of predictors in the structural model specification, that is, the violation of the exogeneity assumption in dependency models.

Most of these situations are associated with the common unreflective use of statistical packages or with the old saying: “If all you have is a hammer, everything looks like a nail.” From now on, as it is previous to any conclusion or even data analysis process, we name this error as *the ritualistic error or type zero error*.

### The illustrative Kolb's experiential learning theory case

**Figure 1:**
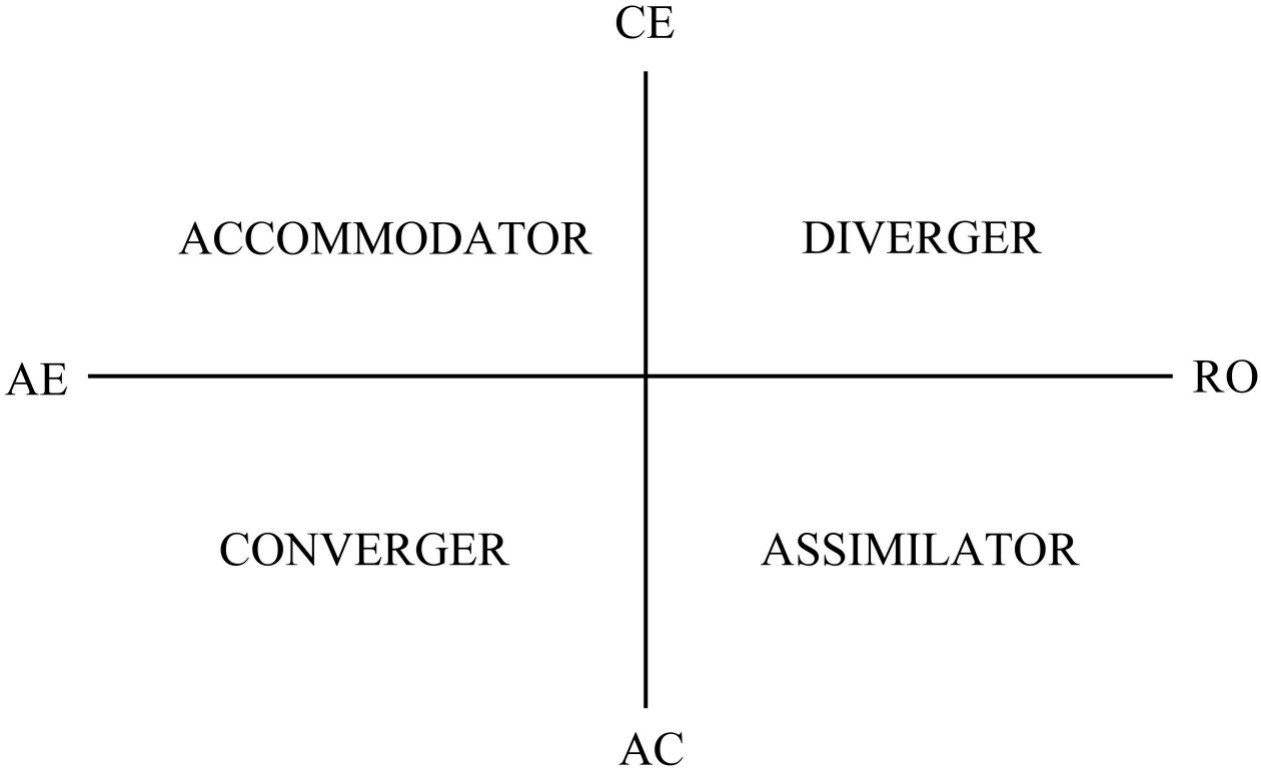
**ELT learning modes and styles; prehension in the vertical axis and transformation in the horizontal axis**.

Let us illustrate this omnipresent *Zero error* within Kolb's Experiential Learning Theory (ELT), which defines learning as “the process whereby knowledge is created through the transformation of experience, knowledge results from the combination of grasping experience and transforming it” (Kolb, 1984, p. 41). According to ELT, people learn through an iterative process with four modes: abstract conceptualization (AC), concrete experience (CE), reflective observation (RO), and active experimentation (AE; Kolb, 1984; Kolb and Kolb, 2015), which are assessed through the Kolb Learning Styles Inventory (KLSI, Kolb, 1985/1999; Kolb and Kolb, 2013). These modes are organized around two dialectical tensions: comprehension-apprehension (AC-CE) and extension-intention (AE-RO). It appears that people have a preference for some combination, often of two learning modes, which are called learning styles (LS): The CE and AE combination is an *Accommodator* style; AE and AC, a *Converger* style; AC and RO, an *Assimilator* style; and CE and RO, a *Diverger* style. These LS are often portrayed on a two-dimensional graph, as shown in Figure 1 (Kolb, 1984). In the last 10 years, patterns of learning preferences have helped to identify nine learning styles: the four above plus a Northern style, predominantly CE; a Southern style, predominantly AC; an Eastern style, predominantly RO; a Western style, predominantly AE; and a balance of all four.

The dialectical dimension that represents the opposition between information apprehension through the concrete experience mode (CE) and the abstract conceptualization mode (AC), which leads to information comprehension, is known as *prehension*. The other dimension is called *transformation*. Transformation occurs through the conversion of information into knowledge from the reflective observation mode (RO), which involves a more internal process of intention, to the active experimentation mode (AE) through a process of extension.

Kolb defines LS as preferences for some modes over others. To achieve *ecological validity* (Brunswik, 1943), Kolb's operationalization of the four-mode construct specifies two opposite pairs or dimensions of learning modes. Consequently, KLSI forces respondents to prioritize among the four alternative modes instead of presenting them as four independent factors. This forced choice format of the KLSI produces constraints on the primary mode data within a fixed sum, which are ipsative data. Research questions about the ipsativity of the LS have resulted in a focus on the two dialectical dimensions (Kolb and Kolb, 2005, 2013), which has resolved many of the statistical aberrations and has led to a more convenient approach to the study of learning styles (Kolb and Kolb, 2005, 2013)^1^.

Since the beginning, the artifactual negative correlations in the KLSI due to the forced-ranking effect were clearly identified (Kolb and Kolb, 2005, 2013), and some researchers proposed possible corrections to remove this effect from the KLSI (Certo and Lamb, 1980). “The use of ipsative measurement in social science research remains controversial […] problems with the correct understanding of the results, and complaints by respondents that it is very hard to fill out questionnaires containing forced-choice items are well-known dilemmas […] to start solving the problem of the statistical analysis […] is an essential first step to put an end to the long-lasting controversy about the use of ipsative measures in scientific research” (van Eijnatten et al., 2015, p. 574). There have been efforts to address ipsativity in the KLSI: “Ipsative measures can, under certain conditions, be effectively transformed to non-ipsative ones” (Mainemelis et al., 2002, p.10). The common practice has operationalized the first two dialectical dimensions by subtracting the opposing pairs, AC-CE (prehension) and AE-RO (transforming). In the KLSI, the subtracted mode scores, AC-CE and AE-RO, are truly non-ipsative. Notwithstanding, the subtracted scores are bounded, so they violate the usual assumption of any model with an unbounded (e.g., normal) distribution of the error term, which is a type zero error. Fortunately, unique solutions for the statistical analysis of ipsative data have been developed, in quite another discipline in what is known as compositional data analysis—CODA (van Eijnatten et al., 2015). Like these authors, we have been very much inspired by those solutions, because compositional data are a particular case of ipsative data.

This article is organized as follows. We first present the alternative CODA method for analysing theories whose faithful operationalization leads to compositional data, as the case is for the KLSI. In short, CODA transforms the data into an unbounded space and applies standard statistical methods on the transformed data. We show how this new analytical approach leads to the geometrical existence of a third dimension on the KLSI, which relates the two dialectical dimensions—prehension and transformation of experience. Then, in an attempt to provide a better understanding of this third dimension we test its relationship with other external variables looking for criteria validity evidences. Next we illustrate it with real data from full time MBA participants in an Emotional and Social Competencies development program where ELT is used as part of the learning plan design process. We finally come to the conclusions and discussion.

## Methods

### Compositional data as a particular case of ipsative data

Unfortunately, this mentioned knowledge of the interdependent nature of the data that KLSI produces did not preclude both advocates and detractors of the ELT from too frequently basing their pros or cons analyses on Classical Test Theory or basic econometrics. This assumes that the KLSI learning modes have independent unbounded errors (Yahya, 1998; Kolb and Kolb, 2013).

However, the KLSI actually conforms a particular and interesting case of ipsative data, which are called compositional data. Compositional data are interesting to researchers as carriers of information about the relative importance of components (in our case, learning modes) for each individual. Compositional data have a zero lower bound and can be represented with a fixed sum (Pawlowsky-Glahn et al., 2015). The KLSI contains 12 items in which respondents rank the four modes which may be coded from 0 (the least preferred) to 3 (the most preferred). The sum of rankings of each mode across the 12 items ranges from 0 to 36 and has a fixed 72 sum. Then KLSI can be understood as an instrument that distributes 72 points among the four learning modes, which are the four components.

Thus, the score of a mode is the number of times that mode is preferred over any other mode in all possible pair-wise comparisons in all 12 KLSI items. For instance, if a mode is always ranked as the lowest it has never been preferred to any other mode and gets a 0 score. If a mode is always ranked as the highest, it is preferred 12 times to the three other modes and gets a 36 score. Scores can thus be understood as having ratio scale properties: a mode with score equal to six has been preferred twice as many times to other modes across the 12 items than a mode with score equal to three.

For convenience, the scores can be divided by 72, thus revealing the proportion of the four modes used by each individual. Proportions between 0 and 1 are the most common way to present compositional data.

One feature of compositional data is the bounded distribution of scores, which theoretically makes the usual modeling using unbounded probability distributions inappropriate, although in some instances, the results have been found to be relatively robust. More importantly, the usual statistical modeling does not fully utilize the potential of the relative information carried by this type of data. For instance, the relative importance of one component can increase only if another decreases. This property prevents the interpretation of the effects of linear models in the usual way by keeping everything else constant.

CODA was developed by Aitchison (1986) as a means of treating data from chemical and biological analyses. In chemical and biological compositions, data have a relative nature and the interest of the researchers is only or primarily the relative importance of chemical or biological components. CODA has the twofold objective of making compositional data statistically treatable and of getting the most of the relative information carried by the data. Social scientists have recently become aware of the CODA tradition, and have successfully used it when faced to similar problems (e.g., Coenders et al., 2011; Fry, 2011; Kogovšek et al., 2013; Ferrer-Rosell et al., 2015, in press; Trendafilov and Gallo, 2015; van Eijnatten et al., 2015; Vives-Mestres et al., 2015). The problem of possible fixed sum representation and the interest in relative information are shared by KLSI researchers who can thus benefit from using CODA.

The simplest CODA methods propose the transformation of compositions by means of logarithms of ratios to both overcome the distribution boundary issue and focus the analysis on relative rather than absolute differences. It is well-known that logarithms of ratios recover the unconstrained minus infinity to plus infinity range. More importantly, both logarithms and ratios focus on relative importance and on differences between modes in relative rather than in absolute terms. This approach is attractive to applied researchers because once transformed, compositional data can be subjected to standard and well-understood statistical techniques (Aitchison, 1986; Pawlowsky-Glahn and Buccianti, 2011).

Another issue is dimensionality. Given the fixed sum constraint, a four-term composition such as the KLSI actually resides in a 3-dimensional geometrical space called the simplex. Accordingly, it requires three dimensions (in CODA terms, three log ratios) to be represented. In this paper, we indeed show that in our case, in addition to its usual attractive features, CODA “serendipitously” has made the third dimension to emerge. This geometric dimension naturally compares prehension and transformation of experience as a log ratio. Ignoring the existence of this third dimension of the KLSI (so far, just of geometric nature), can only detract from the relationships between the KLSI and other variables. This omission would only be justified if the third dimension fails to exhibit plausible relationships with other substantive variables, or if it fails to be theoretically meaningful^2^.

### Interpretable log ratio transformations for the KLSI

Several log ratio transformations have been suggested in the CODA literature (Egozcue et al., 2003), and recently, there has been a trend toward flexibility and interpretability of the transformations. In general, an interpretable log ratio transformation is easy to compute whenever there is an interpretable sequential binary partition of components into pairs of groups of components based on the conceptual similarity of the components, according to the researchers' objectives, or common research practice. These partitions start by dividing the components into two clusters and then by subdividing one of the clusters into two, until each component constitutes its own cluster. These partitions are best understood as a partition tree or dendrogram (Pawlowsky-Glahn and Egozcue, 2011). An interpretable log ratio transformation simply takes the ratios of the geometric means of the two component clusters at each partition. There is always one fewer log ratio than there are components, and the numerators and denominators are interchangeable. One single component acts as its own geometric mean.

**Figure 2:**
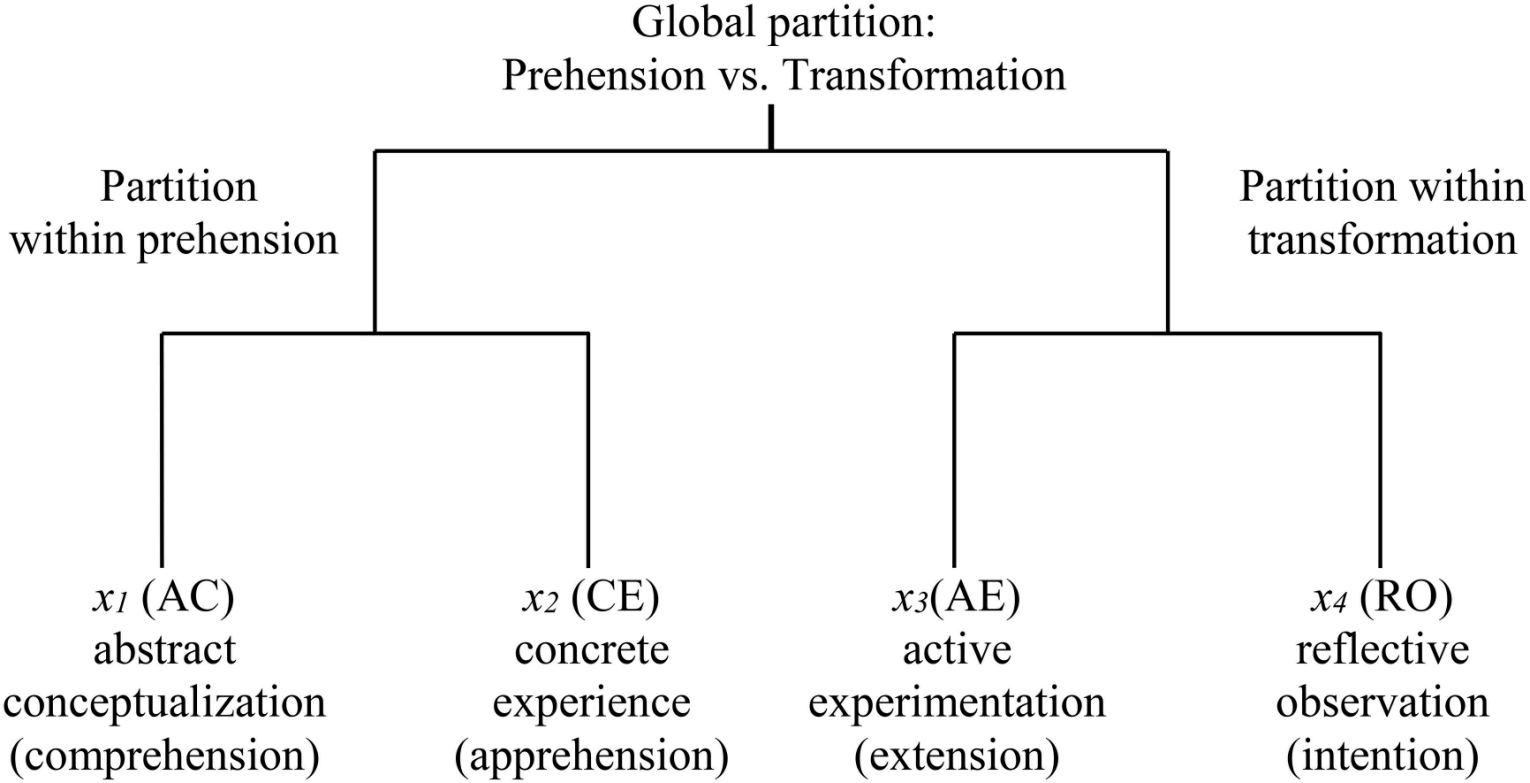
**Sequential partition of modes in the KLSI**.

In the particular case of the KLSI, a meaningful log ratio transformation involves the geometric means of the two groups of modes—prehension and transforming—that dialectically relate the four modes, AC-CE and AE-RO. Figure 2 portrays the four learning modes' partition in a dendrogram, where *x*_1_ = abstract conceptualization-comprehension (AC), *x*_2_ = concrete experience-apprehension (CE), *x*_3_ = active experimentation-extension (AE), and *x*_4_ = reflective observation-intention (RO). *x*_1_ to *x*_4_ are proportions between 0 and 1.

The commonly specified tension between the two pairs of dialectical modes allows us to make a useful interpretation of the following log ratios of the mode proportions. We must take into account that the logarithm of a ratio simply equals the difference between the logarithms.

(1) $$y_{1}=\ln\;\left(\frac{x_{1}}{x_{2}}\right)=\ln\;(x_{1})-\ln\;(x_{2})$$

Equation (1) is the ratio of AC over CE, which can easily be related to the common practice of opposing the modes in a dialectical pair. If *y*_1_ > 0, then AC–CE > 0. In other words, Equation (1) shows the extent to which learners are more abstract conceptualizers (comprehenders) than concrete experiencers (apprehenders), that is, how much more Southern than Northern they are in Figure 1.

(2) $$y_{2}=\ln\;\left(\frac{x_{3}}{x_{4}}\right)=\ln\;(x_{3})-\ln\;(x_{4})$$

Equation (2) is the ratio of AE over RO. If *y*_2_ > 0, then AE–RO > 0. *y*_2_ reveals the extent to which learners are more extensional, active experimenters than intentional, reflective observers, that is, how much more Western than Eastern they are.

These log ratios, *y*_1_ and *y*_2_, jointly make a straightforward characterization of the usual learning styles: a converger, when both *y*_1_ and *y*_2_ are above certain normative values; a diverger, when both *y*_1_ and *y*_2_ are below; an accommodator, when *y*_1_ is below and *y*_2_ is above; and an assimilator, when *y*_1_ is above and *y*_2_ is below.

The normative values derived by Kolb (1976, 1981) for the AC-CE and AE-RO differences cannot be translated into log ratios. Instead, normative values for the log ratios can be obtained from quantiles (e.g., the median) in a large representative dataset in the usual manner.

As previously mentioned, the four modes with a constant sum lie in a three-dimensional geometrical space and thus require three log ratios. Although so far only two dimensions have been considered in ELT, when it is operationalized through KLSI there is an implicit missing log ratio that is clearly shown in Figure 2 and that is the ratio of the geometric mean of the AC and CE pair over the geometric mean of the AE and RO pair. This missing log ratio is represented in Equation (3). It must be recalled that the logarithm of a geometric mean is simply the arithmetic mean of the logarithms.

(3) $$y_{3}=\ln\;\left(\frac{\sqrt{x_{1}x_{2}}}{\sqrt{x_{3}x_{4}}}\right)=\frac{\ln\;(x_{1})+\ln\;(x_{2})}{2}-\frac{\ln\;(x_{3})+\ln\;(x_{4})}{2}$$

This dimension shows that Kolb's instrument is actually tri-dimensional by construction. The CODA methodology always involves using all available dimensions. In this case, the third log ratio is also interpretable. This third dimension describes an individual's relative preference for learning by prehension rather than by a transformation of experience. People with scores on Equation (3) larger than a normative value will be more prone to prehension of information through apprehension or comprehension than to transforming information through intention or extension, and the opposite is true for people with lower scores.

The significance of the relationship of the log ratios with relevant external variables cannot be assessed as usual by means of correlations or univariate regression or ANOVA models because compositions are vector variables. Multivariate ANOVA (i.e., MANOVA) models for categorical explanatory variables and multivariate ANCOVA (i.e., MANCOVA) for continuous explanatory variables are appropriate. In these models the three log ratios simultaneously act as dependent variables. MANOVA and MANCOVA multivariate tests and statistics (e.g., Pillai's trace, Hotelling's trace, or Wilk's Lambda) are invariant to how components are arranged in the partition tree leading to the log ratio choice (Mateu-Figueras et al., 2013). Of course, univariate tests that refer to each particular log ratio are not invariant, hence the importance of the interpretability of each log ratio. Apart from this interpretational issue, once the log ratios have been built, the use of MANOVA and MANCOVA is standard in all respects because the proposed log ratios for the KLSI are fully interpretable.

### Sample

In this study, the data collection covered seven consecutive years (2006–2013) of candidates at an international MBA program in ESADE BS, a European Business School. The sample size was 1194 full-time MBA participants with 86 different nationalities, of which the most common were Spain (15.9%), the US (13.7%), India (9.6%), Germany (5.6%), Mexico (4.3%), Brazil (4.1%), Japan (3.5%), Canada (2.9%), Italy (2.8%), Colombia (2.7%), and Portugal (2.2%). Males composed 69.7% of the sample; females, 30.3%. The average age was 31.4 years (SD 2.8 years), and 96.7% of participants were aged between 26 and 37. The students' educational backgrounds were also heterogeneous, including not only economics (11%) and management (32%) but also engineering (36.4%), social sciences (4.3%), law (3.5%), hard sciences (5.5%), arts (5.7%), and psychology (1.5%).

### Measures

As part of the MBA curriculum, which is taught in English, students participate in a Leadership Development Program based on Intentional Change Theory (Boyatzis, 2008). The program is conducted via a digital platform that provided the data used in this research. Participants provide informed consent and at any moment they can complete any of the exercises or tests in confidential mode, in which case the data are excluded from all analyses.

The program includes the evaluation of the learning styles using the KLSI. As previously mentioned, the data were then recoded to a 0-1 range to obtain the *x* variables^3^. Log ratios (*y*) were then computed as in Equations (1–3).

Regarding external variables, as part of the course, students were evaluated using the Emotional and Social Competency Inventory—University Edition (ESCI-U; Boyatzis and Goleman, 2007), which is a 70-item survey instrument that measures 14 personal competencies divided into two types: one with 12 emotional and social competencies and the other with two cognitive competencies (Boyatzis and Goleman, 2007; Boyatzis, 2009). The scales show appropriate convergent and discriminant validity and a good model fit (Boyatzis et al., 2014). Because the behavioral manifestations of these competencies are frequently observed in a variety of different situations, they have been operationalized using as many as five indicators per competency. It is believed that multi-source assessment, such as 360°, provides protection against social desirability and common method bias because of the distinct sources. Assessments were averaged over raters who came from the students' personal and professional circles without taking into account self-evaluations. The 14 competencies were grouped into five theoretical clusters: self-awareness, social awareness, self-management, relationship management, and cognitive competencies. All competencies were scored from 0 to 10 (Batista-Foguet et al., 2009).

**Figure 3:**
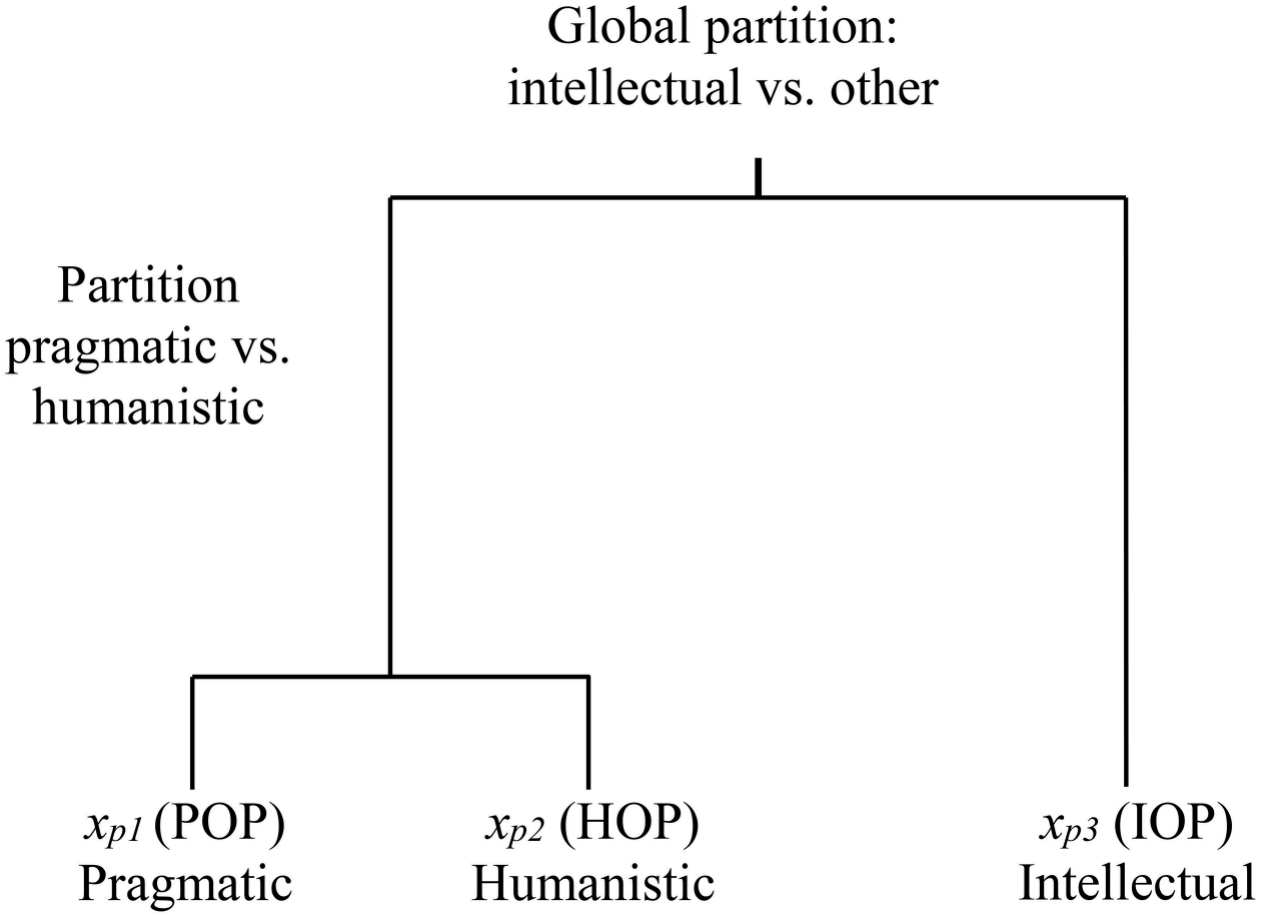
**Sequential partition of philosophical orientations**.

Continuing to the external variables, participants completed the Philosophical Orientation Questionnaire (POQ). Classical and current philosophy suggests three major value criteria (orientations) of philosophical systems that describe the extent to which a person is pragmatic, intellectual, or humanistic (Boyatzis et al., 2000). The central theme of the first, the pragmatic operating philosophy (POP), is the belief that the determination of utility provides a measure of worth. The central theme of the second, the intellectual operating philosophy (IOP), is the attempt to make sense of the world by constructing an image of it and how it works. People with a predominant intellectual philosophy rely on intellect and logic in making decisions. The central theme of the third, the humanistic operating philosophy (HOP), is that personal relationships create meaning in life. People with a predominant humanistic philosophy are assumed to be committed to human values, particularly those that stress relationships as the highest good. In Boyatzis's POQ, respondents are asked to rank three available choices in each of 20 questions, with each choice reflecting an orientation. The questionnaire is thus ipsative and provides again compositional data on the relative importance of the three orientations. We note the components as *x*_*p*__1_ = pragmatic (POP), *x*_*p*__2_ = humanistic (HOP), and *x*_*p*__3_ = intellectual (IOP). We use subscript p merely to identify variables related to philosophical orientation rather than learning styles. Figure 3 shows the philosophical orientation dendrogram, which is used to compute the log ratios. Although there are other possible ways of partitioning philosophical orientations in the dendrogram, the overall tests are invariant to this choice, as stated in the CODA methodology section.

The first log ratio compares the intellectual component with the geometric mean of the pragmatic and humanistic philosophies. Positive values reveal an intellectual share greater than the geometric mean of the remaining two philosophy components. Negative values reveal the opposite.

(6) $$\begin{array}{l} y_{p1}=\ln\;\left(\frac{x_{p3}}{\sqrt{x_{p1}x_{p2}}}\right)=\ln\;(x_{p3})-\frac{\ln\;(x_{p1})+\ln\;(x_{p2})}{2} \end{array}$$

The second log ratio is the ratio of the pragmatic orientation over the humanistic. Positive values reveal a pragmatic share that is larger than the humanistic. Negative values illustrate the opposite.

(7) $$y_{p2}=\ln\left(\frac{x_{p1}}{x_{p2}}\right)=\ln\left(x_{p1}\right)-\ln\left(x_{p2}\right)$$

In our dataset, the philosophical orientations contained no zeros.

## Results

### Three dimensions of learning

Table 1 shows the four LS mode components and the three log ratios. The relative magnitude of the components' geometric means show that, as a whole, students are more Southern than Northern and more Western than Eastern in Figure 1. These results are aligned with those obtained from 1286 MBA students by Kolb and Kolb (2005). Means and medians of components are advised against because they do not follow the geometry of compositional data. Geometric means are a far better location measure for compositional data (Pawlowsky-Glahn and Egozcue, 2002).

**Table 1:** **Description of mode components (***x***_***d***_) and log ratios (***y***_***d***_)**.

	**Min**.	**Max**.	**Median**	**Mean**	**Geometric mean**	***SD***
**LEARNING MODE PROPORTIONS**						
*x*_1_ abstract conceptualization-comprehension (AC)	0.042	0.500	0.306	0.305	0.285	0.100
*x*_2_ concrete experience-apprehension (CE)	0.009	0.472	0.167	0.178	0.154	0.088
*x*_3_ extensional active experimentation (AE)	0.028	0.500	0.306	0.305	0.287	0.095
*x*_4_ intentional reflective observation (RO)	0.009	0.472	0.208	0.212	0.184	0.094
**LOG RATIOS**						
*y*_1_ log ratio AC over CE	−2.335	3.918	0.580	0.617	—	0.834
*y*_2_ log ratio AE over RO	−2.428	3.789	0.414	0.442	—	0.823
*y*_3_ log ratio prehension over transformation	−1.676	2.085	−0.116	−0.095	—	0.538

As regards log ratios, means and medians are appropriate location measures. The medians of the log ratios serve as examples of how to obtain normative values, taking into account that the available sample makes it risky to assume these median values can be used beyond this particular case.

To interpret the range of the log ratios, it must be taken into account that a log ratio equal to 1 indicates that the numerator is exp(1)≅2.72 times larger than the denominator, a log ratio equal to 2 indicates that the numerator is exp(2)≅7.39 times larger than the denominator, etc. Negative log ratios of the same magnitude show the same relative sizes when the denominator is larger than the numerator.

Regarding variability, a log ratio with little or no variability indicates that the components in the numerator and denominator increase together so that there is no variation in their relative size. If the third log ratio (dimension) were completely unimportant, then prehension and transformation would have equal relative sizes for all individuals, and the log ratio would have a zero standard deviation.

Regarding the distribution of the log ratios, while it tends to be unbounded, it is not necessarily normal. The normality hypothesis is in fact rejected at 1% significance by the Shapiro–Wilk's test for all three log ratios. This is hardly surprising given the fact that the large sample size provides the test with a high statistical power. Fortunately, the same large sample size guarantees the robustness of the key MANOVA and MANCOVA results under non-normality.

The first two log ratios reveal opposite learning modes and have a large standard deviation, as expected. Although the standard deviation of the third log ratio is smaller than that of the first two, it nonetheless reveals substantial differences among individuals. In the sample, it is easy to find log ratios equal to 1 or -1 and thus to find students who show 2.72 times more of a prehension orientation than transformation, or the other way around. Far from being pure dialectical opposites, large values of both AC and CE can coexist in certain students, who are referred to by Mainemelis et al. (2002) as *mixed types*; similarly, there may be people with large values of AE and RO, which demonstrates a large transformation orientation. For instance, a person with a negative *y*_3_ score would, on the whole, have higher AE and RO scores and lower AC and CE scores and thus have more of a transformation orientation than prehension. The sign of *y*_2_ tells us this person's preference for transforming knowledge in a particular way.

### The three dimensions and philosophical orientations

Each philosophical orientation log ratio had a highly significant effect on the dimensions of the KLSI as a whole according to the MANCOVA model global tests (Pillai's trace, Hotelling's trace, and Wilk's Lambda yielded identical results). *F*-tests showed that the relationship between philosophical orientation as a whole and *y*_1_ and *y*_3_ was significant, but not the relationship with *y*_2_. We retained *y*_2_ in the interpretation because it was individually significantly related to *y*_*p*__1_.

As shown in Table 2, the results regarding the first two learning-style log ratios correspond to the findings of Boyatzis et al. (2000). An IOP is positively related to *y*_1_, which indicates an emphasis on AC, negatively related to *y*_2_, which indicates an emphasis on reflective observation, and positively related to *y*_3_, which emphasizes prehension over transformation. The strong link between IOP and *y*_3_ adds to the understanding of learning preferences: IOP is related not only to AC but to prehension as a whole.

**Table 2:** **Effects from philosophical orientation log ratios on learning style log ratios and joint tests' ***p***-values**.

	**Pillai's trace *p*-value**	***y*_1_: Prehension: comprehension (AC) over apprehension (CE)**	***y*_2_: Transformation: extension (AE) over intention (RO)**	***y*_3_: Prehension over transformation**
IOP over other (*y_*p*_*_1_)	0.000	0.307^**^	−0.106^*^	0.109^**^
POP over HOP (*y_*p*_*_2_)	0.000	0.220^**^	−0.008	−0.089^**^
*F*-test *p*-value		0.000	0.138	0.000

* Statistically significant at 5%;

** *statistically significant at 1%*.

A pragmatic compared with a HOP shows an emphasis of POP on *y*_1_ toward AC and a negative *y*_3_ toward transformation over prehension as a whole. However, its strong link to AE in prior research (Boyatzis et al., 2000) is only part of the story in this position on *y*_3_. The portrayal of HOP on the three dimensions is also consistent with prior results: HOP is emphasized with CE and prehension.

### The three dimensions and emotional and social competencies as seen by observers

In this section, we provide a stronger test of the third dimension because the data come from two different sources. As previously mentioned, learning dimensions were obtained via self-assessment, whereas the competency information was provided by observers. The raters' perception of each cluster of social and emotional competencies had a highly significant relationship with the dimensions of the KLSI as a whole according to the MANCOVA model global tests (Pillai's trace, Hotelling's trace, and Wilk's Lambda yielded identical results). *F*-tests showed the relationship between emotional competencies as a whole and *y*_1_, *y*_2_, and *y*_3_ to be significant.

Table 3 shows that the KLSI dimensions have significant relationships with the ESCI-U clusters of competencies as perceived by others. Starting with the first competency (first row from Table 3), as other researchers we have also found that emotional self-awareness is associated with CE (Boyatzis et al., 2000). The finding that it was negatively related to y_1_ confirms that people with the self-awareness competency would tend to be more likely to grasp information through CE than through AC. All other results are also plausible. Self-management is negatively loaded on *y*_3_, thus emphasizing transforming. Social awareness is also negatively related to *y*_3_, thus emphasizing transformation as a whole, and its negative relationship with *y*_2_ emphasizes that they tend to use RO over AE within transformation. Relationship management is positively loaded on *y*_3_ with an emphasis on prehension as a whole, and within prehension, it has heavier CE than AC, as indicated by its negative loading on *y*_1_. In addition, its positive loading on *y*_2_ places the emphasis on AE more than on RO. Finally, the cognitive cluster is positively loaded on *y*_3_, thus emphasizing prehension as a whole, and as expected, it shows a positive association with *y*_1_ emphasizing AC over CE.

**Table 3:** **Effects from theoretical clusters of social and emotional competencies on learning style log ratios and joint tests' ***p***-values**.

	**Pillai's trace *p*-value**	***y*_1_: Prehension: comprehension (AC) over apprehension(CE)**	***y*_2_: Transformation: extension (AE) over intention (RO)**	***y*_3_: Prehension over transformation**
Self-awareness	0.024	−0.150^**^	0.063	0.010
Self-management	0.044	0.101	0.019	−0.156^**^
Social awareness	0.018	−0.022	−0.223^**^	−0.112^*^
Relationship management	0.000	−0.378^**^	0.261^**^	0.195^**^
Cognitive competencies	0.000	0.467^**^	−0.067	0.092^*^
*F*-test *p*-value		0.001	0.004	0.000

* Statistically significant at 5%;

** *statistically significant at 1%*.

A more detailed analysis of the third dimension shows that the positive pole of prehension is associated to cognitive competencies and to relationship management. Within this positive pole, as expected, the cognitive cluster is related to comprehension (the realm of thought), whereas relationship management is related to apprehension (the realm of feelings). Regarding the transformation pole of the third dimension, it is related to social awareness and self-management, with the former tending to transform through intention (RO) and the latter showing no particular preference for either mode of transforming.

## Discussion

By using the CODA approach, we distilled the information about the relative size of the learning modes, as carried by the ipsative KLSI instrument. At the same time, we resolved the ipsativity problem. The technique we adopted is well-known in other scientific fields that face similar problems and starts to be used in the social sciences. The approach computes the log ratios of the components before applying standard statistical models. The log ratios are built with the aid of the dendrograms in Figures 2, 3. In this manner, learning styles can be related to numeric variables (e.g., competencies) and even to other ipsative variables (e.g., operating philosophies).

The first two dimensions established by CODA are closely related to the usual two KLSI dimensions, and even the learning styles can be defined based on these “log re-expressed dimensions.” Consequently, they can easily be identified from the previous literature—prehension and transformation. However, using CODA and log transformations, the third dimension of the KLSI was revealed. Three dimensions are not an artifact of the CODA methodology but the natural outcome of the operationalization of Kolb's ELT with four modes that have a fixed sum.

CODA builds the third dimension as one that describes an individual's relative preference for learning by prehension (i.e., grasping knowledge) rather than by transformation (i.e., making sense of knowledge). The addition of the third dimension does not modify the first two. Actually, this dimension is conceptually independent from the two prior ones, which leads to two opposite considerations. On the one hand, the fact that *y*_3_ provides exclusively new information makes it very valuable. On the other hand, its novelty makes it difficult to relate this third dimension to the previous literature because the previous literature refers to the first two known dimensions that are unrelated to it.

This third dimension can at best provide new insights into the relationships between learning modes and external variables. Even at worst, it cannot harm the relationships which are found with the first two dimensions. It can be argued that, while in doubt about the third dimension's ultimate importance and implications, it is potentially more harmful to omit it from the analyses (the mentioned type 2 error) than to include it in them. We encourage researchers to replicate and expand our findings, on who is good at prehension and at transformation and to provide new findings, on what good graspers and good transformers are good at.

We have emphasized that this KLSI third dimension has geometrical nature. However, in this article we set out to ascertain the meaning to this dimension within the ELT framework. Premises are, first, that ELT defines learning as a process in which learners ideally end up using all four modes, and second, that these modes are dialectically related by pairs. The original two-dimensional portrait of the ELT assumes this dialectical opposition to hold in that learners cannot combine all possible pairs of modes. AC is combined with AE by convergers and AC with RO by assimilators, but a learning style combining AC with CE (graspers who prefer prehension as a whole) does not fit into the two-dimensional picture.

Given the fact that, when using all available dimensions, we have shown that learners combining AC with CE do exist (as well as those combining AE with RO), and the fact that those more flexible learners (Kolb and Kolb, 2013) can quickly adapt and use any mode, any two learning modes can be dialectically opposed only up to a point. So, research translating learning styles into the best suited university teaching practices (e.g., Terry, 2001; Kolb et al., 2014) has to be extended to include these two new combinations. For instance, the finding that CE learners perform better with analogical models while AC learners perform better when trained with conceptual models (Bostrom et al., 1990) would lead to the conclusion that combining both types of models can be the best practice for graspers as a whole. Lecturers combining real life examples (for AC learners) and questions and answers (for RO learners) can be combined for transformers as a whole (Terry, 2001).

Regarding the relationship of the third dimension with external variables, we have reported the relationship of this third dimension with both variables measured using another self-assessment instrument (the POQ); and variables that were measured using external raters (the ESCI-U); the latter avoid the common method variance effect in the statistical relationships found.

The use of the third dimension significantly clarifies and explains the relationship between operating philosophy and learning preferences. Using only two dimensions, one can understand some of the differences in learning preferences among the three operating philosophies, but the third dimension most clearly captures the distinctions. IOP and HOP are linked to prehension as a whole and POP is linked to transformation as a whole. Although, the link between IOP and prehension is inclined toward AC (as demonstrated by its heavy loading on *y*_1_), and the HOP link to prehension is in the opposite direction (toward CE), the link between POP and transformation only appears in *y*_3_, and the loadings on the two prior dimensions do not show a preference for AE or RO.

With regard to competency clusters, as seen by observers, the third dimension showed social awareness with an inclination toward transformation in general and even more so toward RO in particular (negative loading on *y*_2_); relationship management had a tendency toward prehension and even more so toward CE (negative loading on *y*_1_); and the cognitive cluster tended to prehension and even more so to AC. However, self-management, which shows a preference for transformation, does not show any preference for one transforming mode over the other. More importantly, with only the first two dimensions, no relationship between self-management and learning styles was found. This preference of self-management toward transformation most likely emerges from a pragmatic orientation used when people act with these emotional intelligence competencies.

## Conclusion

We have pointed out that ipsative data in general, and compositional data in particular, are often not properly analyzed even when their constraint problems have long been known. We have shown that the data analysis method not only affects numerical results but also has deeper theoretical implications. In this article we have illustrated this using the KLSI but many social sciences measurement instruments provide compositional data that is not even identified as such. Actually, we have dared to give a name to this so pervasive type of error—type zero error. We have shown how using CODA and log ratio transformations all ipsativity concerns are solved while all the relevant information about the relative importance of components is taken into account. Moreover, compositional data can be analyzed using standard statistical techniques. A third dimension of the KLSI was uncovered providing the capability to extend the understanding of learning preferences and to relate them with competencies and operating philosophies. Now we know something about who is good at grasping and who is good at transforming. Much more needs to be learned, including what good graspers or good transformers are good at. We hope that this study opens the door to more research on the learning style preferences and shade some light in the potential benefits of using CODA for analyzing KLSI data in particular, and compositional data in general.

### Conflict of interest statement

The authors declare that the research was conducted in the absence of any commercial or financial relationships that could be construed as a potential conflict of interest.

## Footnotes

^1^In the late 1970s and 1980s, a psychometric debate emerged in the experiential literature that caused the KLSI to be revised twice, once in 1985 and again in 1999. In the 1990s, qualitative (Iliff, 1994, cited in Kolb and Kolb, 2013, p. 41) and quantitative (Hickcox, 1990, cited in Kolb and Kolb, 2013, p. 41) reviews focused on the application of ELT. The critiques often focused more on measurement issues in the KLSI than on the theory. Later, the critiques became focused more on the theory than the measures (Hopkins, 1993; Holman et al., 1997; Vince, 1998; Kayes, 2002; all four cited in Kolb and Kolb, 2005, p. 196).

^2^Actually Mainemelis et al. (2002, p.76) suggested a similar third dimension by referring to mixed learning styles as those employed by “emphasizing the dialectically opposed orientations.” A person could focus more on prehension (both AC and CE) and less on transformation (both AE and RO), or the opposite.

^3^If the x variables contain zeros, then log ratios cannot be computed. A frequent assumption in the CODA literature is that a zero represents a component that is present but that is too small to be observed. Once recoded to a 0–1 range, the minimum observable score in the KLSI is 1/72. Martín-Fernández et al. (2003) suggested replacing x = 0 with 0.65 times this minimum:

(4) $$\begin{array}{c} x^{\prime }=0.65∕72 \end{array}$$

Next, non-zero x values must be reduced to preserve the unit sum:

(5) $$x^{\prime }=x\left(1-\sum_{x=0}x^{\prime }\right)$$

This method performs particularly well if the proportion of zeros is below 10% (Martín-Fernández et al., 2011). In our dataset, the CE and RO modes contained only 0.38% zeros, and the remaining two modes contained no zeros. Alternatives are discussed in Martín-Fernández et al. (2015).
